# Slow saccades in cerebellar disease

**DOI:** 10.1186/s40673-018-0095-9

**Published:** 2019-01-17

**Authors:** Kelsey Jensen, Sinem Balta Beylergil, Aasef G. Shaikh

**Affiliations:** 1 Neurological Institute, University Hospitals, Cleveland, OH USA; 2 Department of Neurology, Case Western Reserve University, Cleveland, OH 44022 USA; 3 Neurology Service, Louis Stokes Cleveland VA Medical Center, Cleveland, OH USA

**Keywords:** Degenerative disorder, Dysmetria, Burst neurons, Reciprocal innervation

## Abstract

Eye movements are frequently considered diagnostic markers indicating involvement of the cerebellum. Impaired amplitude of saccades (saccade dysmetria), impaired gaze holding function (horizontal or downbeat nystagmus), and interrupted (choppy) pursuit are typically considered hallmarks of cerebellar disorders. While saccade dysmetria is a frequently considered abnormality, the velocity of saccades are rarely considered part of the constellation of cerebellar involvement. Reduced saccade velocity, frequently called “slow saccades” are typically seen in a classic disorder of the midbrain called progressive supranuclear palsy. It is also traditionally diagnostic of spinocerebellar ataxia type 2. In addition to its common causes, the slowness of vertical saccades is not rare in cerebellar disorders. Frequently this phenomenology is seen in multisystem involvement that substantially involves the cerebellum. In this review we will first discuss the physiological basis and the biological need for high saccade velocities. In subsequent sections we will discuss disorders of cerebellum that are known to cause slowing of saccades. We will then discuss possible pathology and novel therapeutic strategies.

## Background

Saccades are rapid, simultaneous movements of both eyes. Their purpose is to position the fovea at an object of interest in order to bring the object of interest more clearly into the line of sight. Saccades come in several different flavors, including visually guided (directed towards an intended target), reflexive (directed towards an unexpected target), memory guided (directed toward a remembered location) and antisaccades (equal amplitude movements in the opposite direction of a target). The complex circuitry of visually guided saccades is extensively studied, making it a useful tool for localizing and understanding neurological lesions. A variety of pathologies can alter the size, timing and speed of these saccades. In particular, characterizing changes in the horizontal and vertical velocity of visually guided saccades in various disorders holds promise as an important tool in both the diagnosis and probing treatment outcomes of these disorders. Here we review whether disorders affecting the cerebellum influence the velocity of visually guided saccades. First we will present the basic outline of anatomical and physiological principles underlying the generation of visually guided saccades. The review will then delve into discussion of various cerebellar disorders that are associated with slowing of saccades. We will then compare slow saccades in cerebellar disorders with that of brainstem and basal ganglia abnormalities. Finally we will present the physiological basis for slow saccades in cerebellar disorders.

### Generation of visually guided saccades

**Fig. 1:**
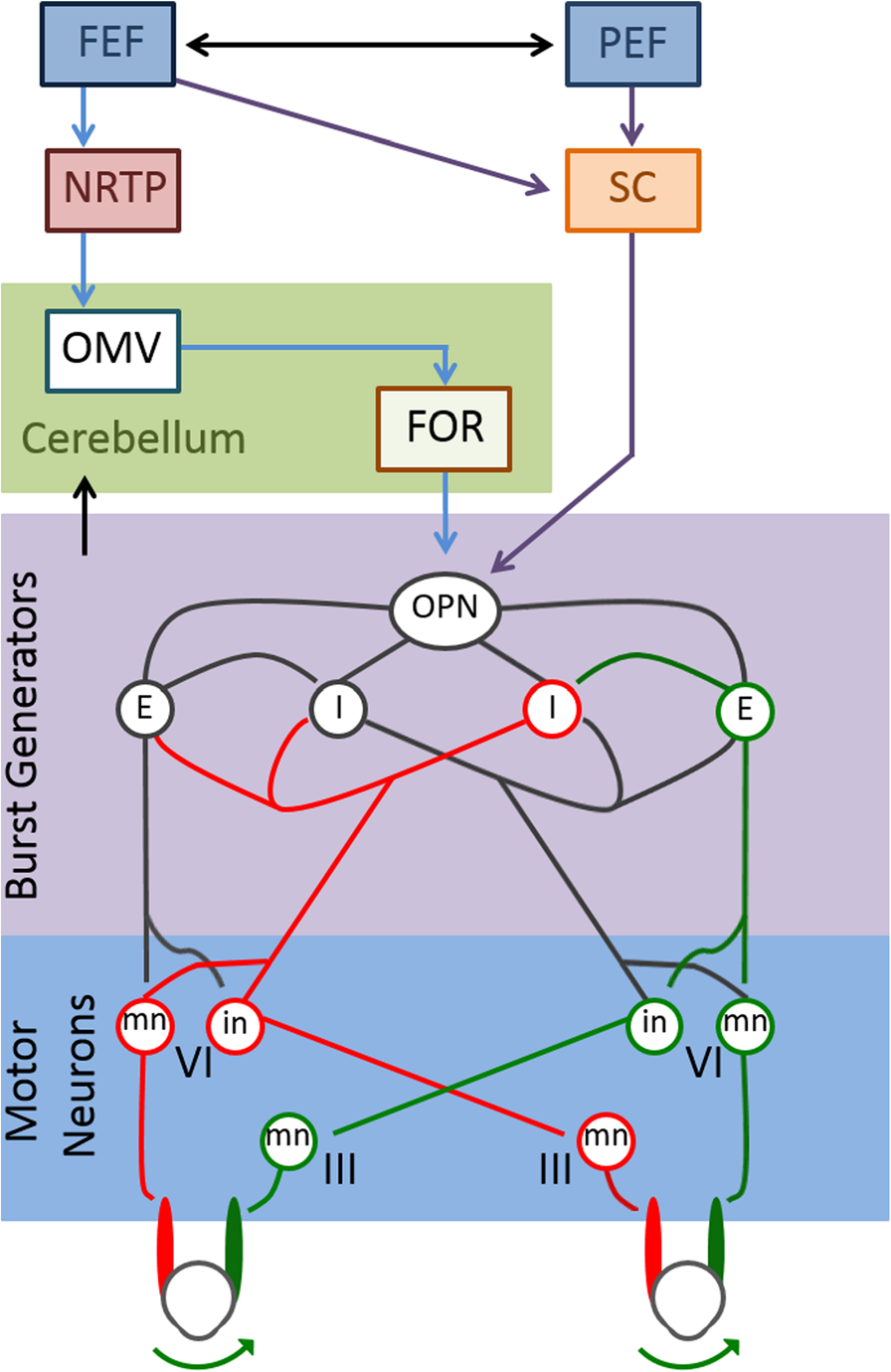
Neural circuitry involved in the generation of visually guided saccades. The frontal eye field (FEF) and parietal eye field (PEF) both send projections to the superior colliculus (SC), which in turn directly communicates with omniopause neurons (OPN) in the midline pons. The FEF also projects to the nucleus reticularis tegmenti pontis (NRTP) in the midbrain. This projects to the oculomotor vermis in the cerebellar cortex, which sends inhibitory fibers to the deep cerebellar fastigial oculomotor (FOR) nucleus. The FOR also communicates with OPN. The OPN is responsible for tonic inhibition of excitatory (E) and inhibitory (I) burst neurons, which results in steady fixation of gaze. When the OPN is inhibited during saccade generation, the excitatory and inhibitory burst neurons fire more rapidly. Excitatory burst neurons synapse on the abducens motoneurons (mn) and internuclear neurons (in) in the ipsilateral abducens nucleus. The internuclear neurons of the side receiving excitatory input project to the contralateral abducens motoneuronsmedial rectus subgroup of the oculomotor nucleus. Ipsilateral inhibitory burst neurons project to the contralateral abducens which inhibits movement of the opposing muscles. This results in the rapid, coordinated movement of gaze to a target object

Visually guided saccade generation begins in the cerebral hemispheres in two major pathways, both of which send input to the superior colliculus (Fig. 1) [1, 2]. In one pathway, the signal originates in the frontal eye field (FEF), supplementary eye field (SEF) and dorsolateral prefrontal cortex (dlPFC) [3–5]. In the other, input originates in the parietal eye field (posterior parietal cortex) [1, 2].

The FEF, SEF and dlPFC send projections to the caudate nucleus, which in turn sends direct, inhibitory fibers to the substantia nigra pars reticulate (SNpr) [6, 7]. The SNpr maintains tonic GABAergic inhibition on the superior colliculus [8, 9]. In sending inhibitory fibers to this structure, the caudate nucleus transiently ceases the firing of inhibitory neurons, leading to timely saccade initiation [10, 11]. The caudate nucleus also sends fibers indirectly to the subthalamic nucleus via the external segment of the globus pallidus [7]. The parietal eye field (PEF) of the posterior parietal cortex projects to the FEF and the superior colliculus. In turn, PEF also receives input from the FEF [12].

The superior colliculus constitutes a shared pathway for visually and memory guided saccades [13]. It integrates excitatory inputs from the cortex and inhibitory inputs from the SNpr [14, 15]. The ventral layers of the superior colliculus consist of a motor map, containing information about eye movement parameters [16, 17]. This structure receives input from the striate, extrastriate, parietal cortex and from the frontal lobes [18, 19]. Electrophysiology studies have also confirmed the presence of neuronal populations directly involved in saccade generation, which project to the midbrain and pontine reticular formation where premotor structures involved in saccade generation are located [20, 21].

The cerebellum receives input both from the cortical eye fields (indirectly via pontine nuclei) and the superior colliculus [22–26]. The dorsal vermis is a medial cortico-nuclear zone within the cerebellum that receives fibers from the nucleus reticularis tegmenti pontis (NRTP) in the midbrain [27]. It projects to the caudal part of the fastigial nucleus, a deep cerebellar nucleus that also receives input from the super colliculus and FEF [26]. This region of the fastigial nucleus, also known as the fastigial oculomotor (FOR), sends projections to omnipause neurons (OPNs), inhibitory burst neurons (IBNs), excitatory burst neurons (EBNs), thalamus, superior colliculus and the reticular formation [28]. FOR modulates saccades by stimulating burst neurons during contralateral saccades and providing inhibition during ipsilateral saccades [29, 30].

The midline pons contains OPNs in the nucleus raphae interpositus [28, 31, 32]. Commands for saccades converge on these OPNs. The superior colliculus directly signals to the OPNs [33–35]. Frontal areas also project indirectly via the caudate nucleus and SNpr [36]. This pathway projects to the cerebellum prior to innervating the OPNs. OPNs are tonically active glycinergic neurons, which produce tonic inhibition of horizontal and vertical saccade burst generators [37, 38]. Suppression of OPNs initiates saccades, whereas electrical stimulation of these neurons stops saccades [39, 40].

EBNs located in several different brain structures have been described. These burst neurons are silent at all times, except during saccades [41]. In the paramedian pontine reticular formation, EBNs relay monosynaptically to oculomotor neurons for horizontal saccadic eye movements [38, 41, 42]. Vertical saccades are generated by EBNs in the rostral interstitial nucleus of the Medial Longitudinal Fasciculus, which is located in the prerubral fields of the dorsal midbrain. The firing rate of EBNs determines the saccadic eye velocity.

Dysfunction in the generation of saccades can be an important clue in determining the site of particular neurological lesions in such multisystem disorders. While pathology involving the eye itself or ocular muscles typically affects the amplitude, velocity and timing of saccades, the central pathology may selectively spare one or more parameters. Thus, understanding the kinematic properties of eye movements can localize lesions found in several neurodegenerative disorders, as well as cerebellar disorders. The readers are referred to previously published resources for the overview and practical guide for examination of the saccades [43, 44].

### Disorders of cerebellum affecting saccade velocity

#### Spinocerebellar Ataxia type 1

Spinocerebellar ataxia type 1 is an autosomal dominant disorder that initially presents with ataxia, but subsequently affects bulbar function including speech and swallowing [45, 46]. Spasticity is also a frequently seen deficit*s* in the spinocerebellar ataxia type 1. The saccade slowing is one of the characteristic features of spinocerebellar ataxia type 1. Although common, unlike spinocerebellar ataxia type 2 (see below), the saccade slowing is not always present in spinocerebellar ataxia type 1 [45]. In addition to slowing of saccades, dysmetria and increased laency is seen in spinocerebellar ataxia type 1 [45]. Since cerebellar degeneration along with brainstem involvement is a  possible structural correlate of spinocerebellar ataxia type 1, it is believed that saccade slowing in spinocerebellar ataxia type 1 could be due to involvement of the brainstem burst generators.

#### Spinocerebellar Ataxia type 2

Spinocerebellar ataxia type 2 is an autosomal dominant disorder due to an unstable expansion of a polyglutamine domain within ataxin-2 [47] which is a cytoplasmic protein found in many body tissues and neurons [48]. The disorder is characterized clinically by progressive cerebellar ataxia, dysarthria, action tremor, early neuropathy, and slowing of saccades [49, 50] . Slowing of saccade velocity strongly correlates with the polyglutamine expansion, but it is inversely correlated with the severity of ataxia [51]. There is no correlation between saccade slowing and the duration of the disease, age of onset or the gender of the diseased subject [51]. Slowing of saccades can be present even before manifestation of other clinical features of the disease [52]. Therefore, saccade velocity is a sensitive and specific marker of disease activity [53] and can be used not only as a surrogate marker of disease severity [54] but also as an early marker for SCA2 [52]. Progression of slow saccades leads to complete gaze palsy in horizontal and vertical directions [55]. Quantitative brain MRI demonstrated reduction in the cerebellar, pons, midbrain, and frontal lobe volumes in SCA2 patients [56]. It is therefore possible that saccade slowing in SCA2 is not primarily a result of cerebellar dysfunction, but it is due to co-existing brainstem abnormality putatively affecting the brainstem burst generation [56, 57]. Indeed, a significant cell loss and reduced synaptic density on somata was found in the mesencephalic area that houses the EBN, which leads to adequate intensity of saccade burst [58].

#### Spinocerebellar Ataxia type 3

Spinocerebellar ataxia type 3, also known as Machado-Joseph disease is an autosomal dominant disorder occurring due to CAG triplet repeat expansion [48, 59–61]. It presents with progressive gait, stance, limb, and truncal ataxia, dysarthria, somatosensory deficits, dystonia and occasionally parkinsonism, dysphagia, and a varying degree of eye movement disorders [62–64]. SCA3 is clinically characterized by a higher frequency of blepharospasm, ophthalmoparesis, nystagmus, increased appendicular tone, and sensory and urinary disturbances [50, 65]. Eye movement abnormalities such as impaired smooth pursuit, optokinetic nystagmus, gaze evoked nystagmus, saccadic dysmetria and horizontal vestibulo-ocular reflex dysfunction are well known deficits of spinocerebellar ataxia type 3 [66–71]. Limitation of gaze in the vertical direction is seen which is more common in the upward direction [72]. Abduction ophthalmoplegia is one of the most common abnormalities in SCA3, however; adduction tends to be persevered [72]. The saccades in some SCA3 patients may have dynamic overshoot of the target, however, those without dynamic overshoot have saccades with low peak velocity [73]. In comparison to SCA2, the inferior olive in SCA3 is rarely affected. Diffuse atrophic changes in the nervous system are observed on MRI with marked changes in the cerebellar vermis, superior cerebellar peduncle, pontine tegmentum, and frontal lobes [74, 75]. Histopathological studies have shown a degeneration of reticulotegmental nucleus of the pons [76] and the omnipause neurons of nucleus raphe interpositus [77]. Degenerative changes in mesencephalic neurons may be responsible for the reduced saccade velocity due to deficient burst generation [77, 78].

#### Wernicke’s encephalopathy

Wernicke’s encephalopathy is caused by a nutritional deficiency of thiamine and is commonly seen in alcoholics. The classic triad of Wernicke’s encephalopathy is ophthalmoplegia, mental confusion, and gait ataxia [79]. Early ocular motor findings in Wernicke’s encephalopathy include gaze-evoked and upbeat nystagmus that may switch to downbeat nystagmus with convergence [80, 81]. Horizontal vestibulo-ocular response impairment occurs early, which progresses to abduction impairment, inter-nuclear ophthalmoplegia, horizontal and vertical gaze palsies, and eventually complete ophthalmoplegia [82–84]. Slowing of saccades is rarely described in Wernicke’s encephalopathy [85]. Wernicke’s disease can progress to Korsakoff’s syndrome, which manifests with a severe memory loss and psychiatric symptoms. Significant eye movement abnormalities include hypometria, slow and inaccurate saccades and impaired smooth pursuit [86, 87]. Patients with Korsakoff’s syndrome also make more directional errors on the antisaccade task [88]. Wernicke’s encephalopathy is not predominantly cerebellar, but like other neurodegenerative conditions also affects extracerebellar brainstem regions including those responsible for the saccade burst generation [81, 89]. It is therefore likely that the slow saccades that are rarely seen in Wernicke’s encephalopathy are a manifestation of impaired saccade burst generation. In atypical forms, Wernicke’s encephalogpathy may affect the substantia nigra [90]. In such instances, impaired saccades could be due to lack of tectal inhibition that is normally provided by the substantia nigra pars reticulata. In such cases, in addition to slow saccades, parkinsonism is also expected.

#### Syndrome of anti-GAD antibody

Glutamic acid decarboxylase (GAD) is an enzyme important in the nervous system for catalyzing the conversion of glutamic acid to γ aminobutyric acid (GABA) – an inhibitory neurotransmitter [91]. Autoantibodies directed against GAD (anti GAD-Ab) have been described in patients with insulin-dependent diabetes mellitus, stiff-person syndrome, epilepsy and in a few patients with late-onset cerebellar ataxia [92–95]. Increased muscle tone, episodic spasms, and cerebellar ataxia are known clinical manifestations [93, 96–99]. Eye movement abnormalities in patients with the syndrome of anti-GAD antibody include downbeat nystagmus, slow vertical saccades, prolonged saccade latency, loss of downward smooth pursuit, saccadic hypometria and dysmetria, impaired ocular pursuit, saccadic oscillations, and impaired cancellation of vestibulo-ocular reflex [100–103]. Case reports of periodic alternating nystagmus [104], and opsoclonus myoclonus [105] have also been described. A study in stiff-person syndrome with cerebellar degeneration described rare saccade abnormalities in the vertical direction. A downward gaze showed multiple hypometric saccades with a normal velocity profile, whereas with upward gaze there was a single saccade with abrupt slowing [102]. We also found saccade slowing in addition to opsoclonus in a patient with syndrome of anti-GAD antibody [103]. Saccade abnormalities in the patient with increased titer of anti-GAD antibody can be multifactorial. For example, frequent interruptions of ongoing saccades, but normal velocity of each “broken” saccade segment suggests impaired programming leading to frequent hypometria, a classic cerebellar phenomenology [106]. Slowing of saccades, however, could suggest involvement of burst generators. An alternate explanation for slow saccades (along with saccadic oscillations) was proposed [103] . Efficient generation of saccades requires not only robust increase in firing rate of the EBNs, latter relies on post-inhibitory rebound [107] . Excessive increase in the excitability due to glutaminergic state (due to immune mediated destruction of the gamma aminobuteric acid (GAD) and decreased degradation of glutamate to GABA) results in baseline increase in the burst neuron excitability. The latter results in an unstable reciprocally innervating circuit of burst neurons, causing opsoclonus, and results in decreased saccade velocity due to lack of the effect of post-inhibitory rebound on hyperexcitable burst neurons. Such physiology, combined, could result in slow saccades and superimposed opsoclonus [108].

##### Can we differentiate various forms of cerebellar disorders based on slow saccades?

While slow-saccades (or their absence) are instrumental, when present, to point out genetic disorders such as SCA 1,2,3, or 6; immune disorders such as syndrome of antiGAD antibody, or infectious disease such as Whipple’s disease; just by itself, the slow-saccades are not able to separate these disorders. Examination of other eye movements, and other co-existing movement disorders are critical in separating one phenomenology from the other even in their classic presentation.

##### Determinants of slow eye velocity in cerebellar disorders

High velocities of saccades depend on an abrupt increase in the excitability of burst neurons due to prompt cessation of the inhibition (the post inhibitory rebound [109]). The sustained inhibition of the excitatory burst neurons is achieved by sustained inhibition of the omnipause neurons, cessation of such inhibition (when saccade is desired) leads to an abrupt increase in excitatory burst neuron firing and rapid velocity of saccades [107–110]. Malfunction of omnipause neurons can cause saccade slowing, but it typically affects vertical and horizontal directions. In contrast, abnormal increases in excitatory burst neuron activity (as expected in syndrome of increased titers of anti-GAD antibody) also reduces the efficacy of omnipause inhibition – hence a slow saccade [103]. Impaired excitability of excitatory burst neurons can lead to deficient burst generation, resulting in slow saccade velocity. Such deficits can be expected in patients with SCA2, SCA3, and Wernicke’s encephalopathy. Maintanence of the saccadic efficiency requires minimizing movement accuracy and learning from the end point error; a key function of the cerebellum. Therefore, disruption of cerebellar function may affect kinematics of saccades including matrix and velocity [57, 111].

It is possible that slowing of saccades in chronic cerebellar conditions has a multifactorial etiology. The structural etiology of slowing, degenerative loss of saccade burst neurons, is one possibility for inefficient velocity command generation. The principles of neuroeconomics determine the basic underpinnings of the second explanation of slow saccade. Disorders of cerebellum, in the presence of uncertainty of destination gaze stability (increased endpoint variability), challenges the brain to optimize saccade accuracy. The optimization takes place in the form of sacrificing the rapidity of movements, hence the slow saccades. Goal directed movements, such as saccades, can occur in infinite possible trajectories onset location to the destination. However, the brain optimizes the trajectory position and speed of voluntary movements (here saccades) to minimize the time-accuracy tradeoff [112] . In other words, the dynamics of eye velocity profiles are determined to make the fastest yet most accurate eye movement and minimal transit time [113]. It was proposed that minimizing the variance of the eye position in the presence of biological and constant noise is the key determinant of trajectory planning of the movement. Minimizing the variability in the eye position in the presence of biological noise is a key determinant of saccade trajectory planning. The noise in the final common pathway that determines the activity of motor neurons leads to deviation of trajectories from the desired path. The deviations are accumulated over the duration of the movement leading to variability in desired position. The noise is independent of the control signal that originally generates the movement [114], however the accumulated error is rapidly minimized by making rapid movement. Rapid movement however requires larger control signals, hence increased variability in the final position. As a consequence the inaccurate movement leads to dysmetria requiring corrective movements [115]. One answer to assure accuracy is low control signals, which leads to slow movements. Thus signal dependent noise imposes trade-off between the movement speed, duration, and the accuracy. It was proposed that the temporal profile of neural command is designed to determine minimal variability in desired position; the velocity of saccade is therefore adjusted accordingly. The end-point variability is inherently higher in patients with cerebellar disorders due to variety of co-existing deficits such as dysmetria, nystagmus, and saccadic intrusions affecting the final gaze position. In the presence of increased endpoint variability, to achieve the best possible endpoint accuracy, the brain compromises on saccade velocity. As a result, the velocity of visually guided saccade is reduced in disorders of cerebellum that frequently causes chronic gaze holding disorders.

##### Multisystem neurodegenerative disorders affecting visually guided saccade velocity

Slowing of saccades can be seen in various conditions that are not prominently cerebellar. For example, visually guided saccades in Parkinson’s disease (PD) patients may have increased prevalence of hypometria compared to healthy controls [116]. Patients with asymmetric PD demonstrated asymmetric hypometria of visually guided saccades and had increased hypometria on the more symptomatic side [117]. This hypometria does not necessarily result in reduced velocity in saccades, but rather increased the time to reach the target by creating a “staircase” trajectory of the saccade in which the gaze is corrected toward the target [118, 119]. Patients with PD have higher variability of peak velocity of visually guided saccades, but saccade slowing is seen only in advanced PD [120].

**Fig. 2:**
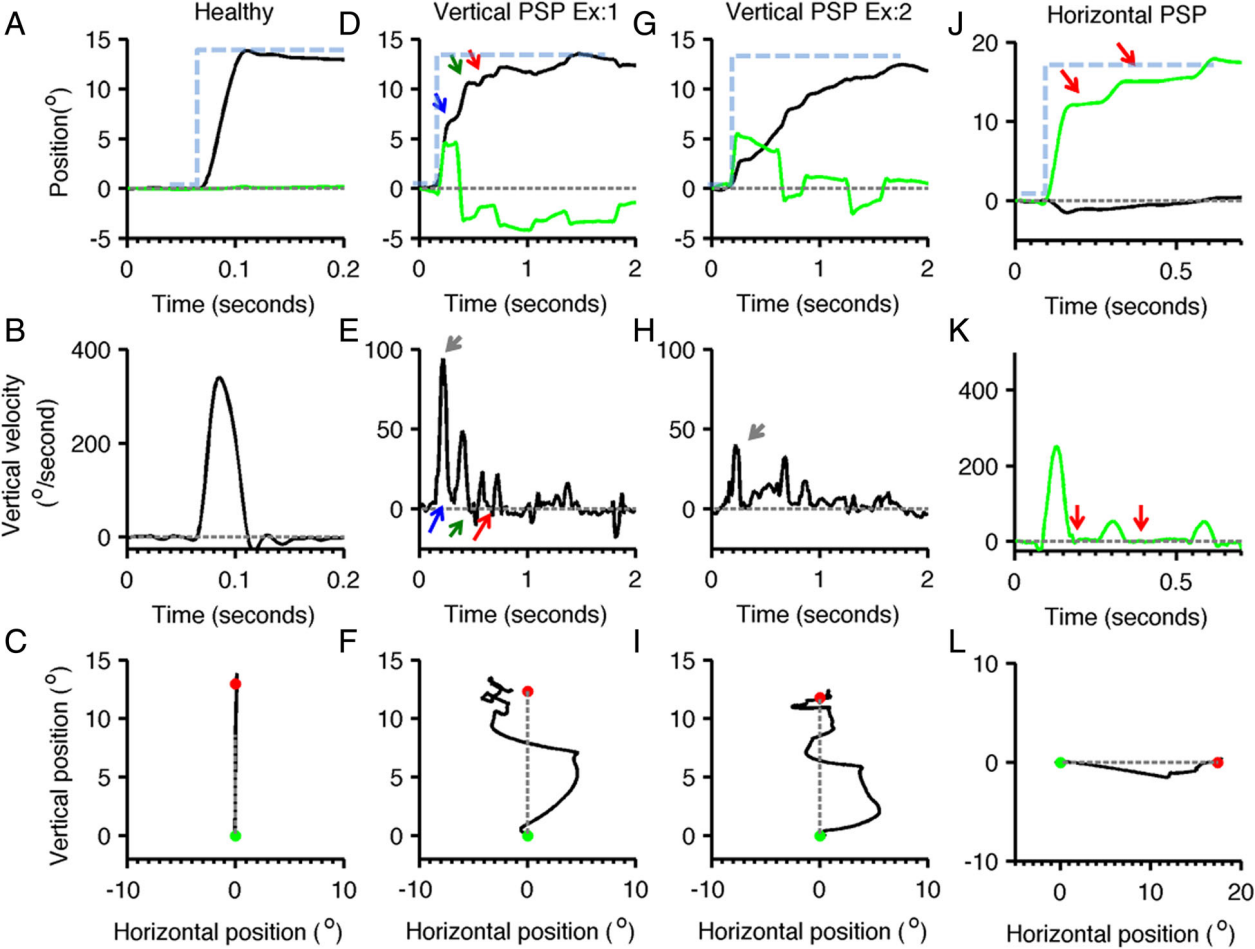
The visually guided saccades from healthy subject and PSP patient are compared. Panel **a** illustrates normal visually guided vertical saccade from a healthy subject. Panels **d**,**g** depict two examples of visually guided vertical saccades from the same PSP subject. Panel **j** depicts eye positions during horizontal saccade. In panels (**a**,**d**,**g**,**j**) the eye position is plotted on the y-axis while x-axis depicts corresponding time in seconds. Black line represents the vertical eye position, while green traces depict horizontal eye position. Grey dashed line is the baseline, i.e. the straight-ahead position, while blue dashed line depicts the desired position. The arrows depict interruption in the saccades, in one type of interruption (blue arrow) the eyes continue to move at slower velocity during interruption, while in other type (green arrow) the slower eye movement in the opposite direction. The third type of interruption (red arrow) is where the eye movements completely stop during the interruption. Panels **b**,**e**, **h**,**k** depict eye velocity. Panel B depicts eye velocity of normal visually guided saccade recorded from the healthy subject, while panels (**e**,**h**) depict vertical eye velocity during vertical saccade in PSP. Green line in panel K illustrates normal horizontal eye velocity during horizontal saccade in PSP. In these subplots the eye velocity is plotted on y-axis while x-axis illustrates corresponding time. Red arrow illustrates interruption in saccade when eye velocity was zero, green arrow is when eye moved at slower velocity in the opposite direction, blue arrow is when eyes moved in the same direction at slower velocity. Panels **c**,**f**,**i**,**l** depict trajectories of horizontal and vertical saccades. Panel C shows the normal saccade from the healthy subject, while panel (**f**,**i**) are vertical saccades in PSP, and panel **l** is the horizontal saccade in PSP. In these plots the green dot depicts the start point, while the red dot is the stop point; grey dashed line is the desired path of the eye movement. Vertical saccades have curved and serpentine path depicting the irregularity in the trajectory. Such curvature is also present in the horizontal saccade but to much lesser extent (adapted from Shaikh et al. [121])

In contrast, slowing of visually guided saccades in either the horizontal or vertical direction may be an important early finding in several of the atypical parkinsonian syndromes. Progressive supranuclear palsy (PSP), a tauopathy that features postural instability, supranuclear gaze and bulbar palsies and axial rigidity, causes saccadic slowing and hypometria (Fig. 2) [121–123]. This change in saccades is more prominent in the vertical axis and with disease progression may result in complete vertical gaze palsy in which vertical saccades are lost [124]. Horizontal saccades are often hypometric early in the course of the disease, and become slower as the disease progresses [125]. Saccades in PSP are not only slow but they also have irregular trajectory [121]. PSP can be distinguished from PD and atypical parkinsonism by a characteristic change in oblique saccades, which have prominent curvature and a relatively faster horizontal component [120].

Neimann-Pick type C disease is another example featuring vertical supraneuclear gaze palsy. This disorder, typically caused by mutations in the NPC1 or NPC2 gene, can lead to paralysis of vertical saccades. It is believed that slowing of vertical saccades in Neimann-Pick type C disease is due to selective involvement of the burst generators at the rostral insterstitial nucleus of Cajal [126].

Slowing of saccades in the vertical and horizontal planes may also be seen in dementia with Lewy bodies [127], cortico-basal degeneration [120, 128] and Huntington’s disease [129–131]. Other changes in saccade parameters and eye movements can also be used to distinguish between these syndromes and may be more characteristic of specific diseases.

The slow saccades are not pathognomonic of mesencephalic disorders, they can be seen in focal, degenerative, or systemic/immune deficits affecting the cerebellum. Cerebellar influence on saccade velocity could be direct, but can be due to altered cerebellar modulation of saccade burst generators or co-existing brainstem involvement in the disorders that primarily affects the cerebellum. Although slowing of saccades provides critical information about the pathophysiology of underlying disease processes, the slowing by itself is not sufficient for diagnostic classification. The latter requires collateral information such as coexisting neurological signs and deficits.

## Conclusion

The classic ocular motor markers of the cerebellar disorder include impairment in amplitude (dysmetria), gaze-holding deficit (nystagmus), and deficit in ocular pursuit. Reduced saccade velocity is considered a usual manifestation of midbrain or multisystem impairment in neurodegenerative disorders such as progressive supranuclear palsy or spinocerebellar ataxia type 2. Decreased velocities of vertical saccades is not rare in cerebellar disorders, it is frequent phenomenology seen in multisystem involvement that substantially involves the cerebellum.

## Abbreviations

Ab: Antibody
dlPFC: Dorsolateral Prefrontal Cortex
EBN: Excitatory Burst Neuron
FEF: Frontal Eye Field
FOR: Fastigial Ocular Motor Region
GABA: Gamma Aminobuteric Acid
GAD: Glutamic Acid Decarboxylase
IBN: Inhibitory Burst Neuron
NRTP: Nucleus Reticularits Tegmenti Pontis
OPN: Omnipause Neurons
PD: Parkinson’s Disease
PEF: Parietal Eye Field
PSP: Progressive Supranuclear Palsy
SCA: Spinocerebellar Ataxia
SEF: Supplementary Eye Field
SNpr: Substantia Nigra Pars REticulata
